# Supplementary material for: Broad CD8+ T cell cross-recognition of distinct influenza A strains in humans
Source: Nat Commun. 2018 Dec 21;9:5427. doi: 10.1038/s41467-018-07815-5 (PMC6303473; doi:10.1038/s41467-018-07815-5)
Supplement: Supplementary file 1 — Supplementary Information [file 41467_2018_7815_MOESM1_ESM.pdf]

Broad CD8+ T cell cross-recognition of distinct influenza A strains in humans

Emma J Grant[1,2], Tracy M Josephs[2], Liyen Loh[1], E Bridie Clemens[1], Sneha Sant[1], Mandvi Bharadwaj[1], Weisan Chen[3], Jamie Rossjohn[2,4,5], Stephanie Gras[2,5#] and Katherine Kedzierska[1#]

[1]Department of Microbiology and Immunology, The University of Melbourne, at the Peter Doherty Institute for Infection and Immunity, Victoria 3010, Australia; [2]Infection and Immunity Program and Department of Biochemistry and Molecular Biology, Biomedicine Discovery Institute, Monash University, Clayton, Victoria 3800, Australia; [3]Department of Biochemistry and Genetics, La Trobe Institute of Molecular Science, La Trobe University, Bundoora, Australia; [4]Institute of Infection and Immunity, School of Medicine, Cardiff University, Cardiff CF14 4XN, United Kingdom; [5]ARC Centre of Excellence in Advanced Molecular Imaging, Monash University, Clayton, Victoria 3800, Australia.

[#]Authors contributed equally.

Supplementary Data summary

Supplementary Figure 1. Cross-recognition of NP_{338} variants after *in vitro* amplification.

Supplementary Figure 2. Gating strategies used for this study.

Supplementary Table 1. Thermal stability of pHLA complexes

Supplementary Table 2. Data Collection and Refinement Statistics

Supplementary Table 3. Donors used in this study and their HLA profiles

Supplementary Table 4. TCRαβ repertoire usage for the recognition of NP_{338}-variant peptides

Supplementary Table 5. Contact table of the EM2 TCR with HLA-B37-NP_{338}

Supplementary Table 6. Surface plasmon resonance (SPR) of the EM2 TCR for NP_{338} and variants

Supplementary Table 7. Conservation of NP_{44} epitope in distinct IAV strains

Supplementary Table 8. TCRαβ repertoire usage for the recognition of NP_{44}-variant peptides

Supplementary Table 9. Primers used in this study

a

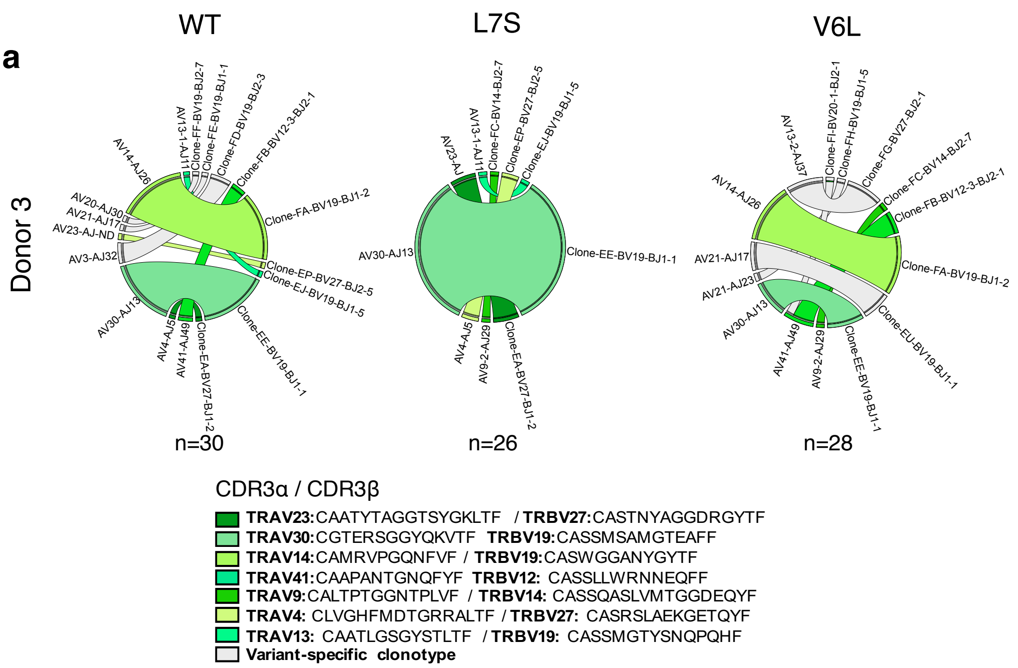

b

Code	TRAV	TRAJ	CDR3α	Length	TRBV	TRBJ	CDR3β	Length	Frequency WT	Frequency L7S	Frequency V6L
EE	30	13	CGTERSGGYQKVTF	9	19	1-1	CASSMSAMGTEAFF	9	33	73	18
FA	14	26	CAMRVPGQNFVF	7	19	1-2	CASWGGANYGYTF	8	33		29
FB	41	49	CAAPANTGNQFYF	88	12-3	2-1	CASSLLWRNNEQFF	9	7		11
FC	9-2	29	CALTPTGGNTPLVF	9	14	2-7	CASSQASLVMTGGDEQYF	13		4	4
EA	23		CAATYTAGGTSYGKLTF	12	27	1-2	CASTNYAGGDRGYTF	10	3	12	
EP	4	5	CLVGHFMDTGRRALTF	11	27	2-5	CASRSLAEKGETQYF	10	3	8	
EJ	13-1	11	CAATLGSGYSTLTF	9	19	1-5	CASSMGTYSNQPQHF	10	3	4	
FD	3	32	CAVRDDLWGGATNKLIF	12	19	2-3	CASSYGTGSADTQYF	10	10		
FE	21	17	CADLPIKAAGNKLTF	10	19	1-1	CASSGVRATEAFF	8	3		
FF	20	30	CAVMVNRDDKIIF	8	19	2-7	CASSIGQAYYEQYF	9	3		
FG	13-2	37	CAESYMGGSSNTGKLIF	12	27	2-1	CASSSLMGGTDEQFF	10			18
EU	21	17	CAVLPIKAAGNKLTF	10	19	1-1	CASSGVRATEAFF	8			14
FH	21	23	CAVMIYNQGGKLIF	9	19	1-5	CASKGDSPHQPQHF	9			4
FI	41	49	CAAPANTGNQFYF	8	20-1	2-1	CSATGTSGYNEQFF	9			4
							**Total # of sequences**		**30**	**26**	**28**
							**Frequency of cross-reactivity**		**83**	**100**	**61**

c

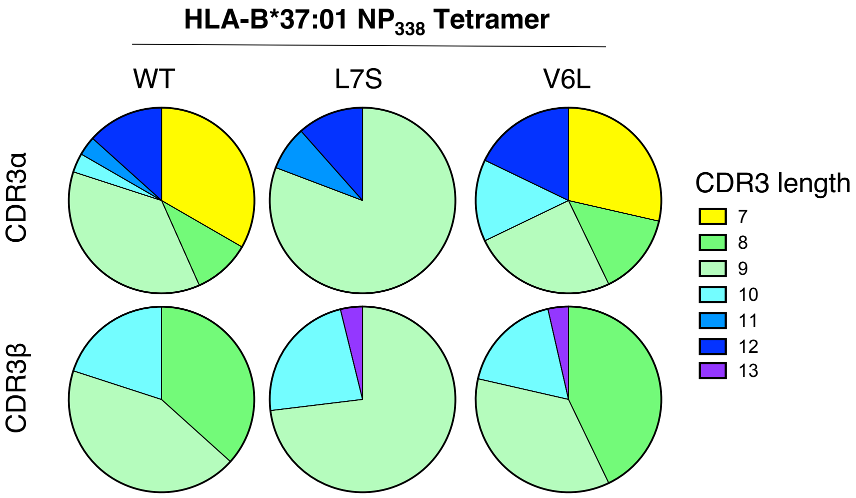

Supplementary Figure 1. Cross-recognition of NP_{338} variants after *in vitro* amplification

PBMCs from HLA-B*37:01^{+} Donor 3 were stimulated with the WT NP_{338} peptide for 10 days. WT NP_{338}-specific CD8^{+} T cell lines were tetramer stained individually with each of the variant NP_{338} tetramers conjugated to PE. Following surface staining, lymphocytes, singlets, live, CD3$^{mid\text{-}high}$ tetramer^{+} CD8^{+} T cells were single cell-sorted and the TCRαβ repertoire was determined using a multiplex RT-PCR. **(a)** Graphical representation and **(b)** summary of the TCRαβ repertoire used by a WT NP_{338}-specific CD8^{+} T cell line for the recognition of the variant NP_{338} peptides. **(c)** Summary of CDR3α and CDR3β length used by CD8^{+} T cells for the recognition of NP_{338} peptides.

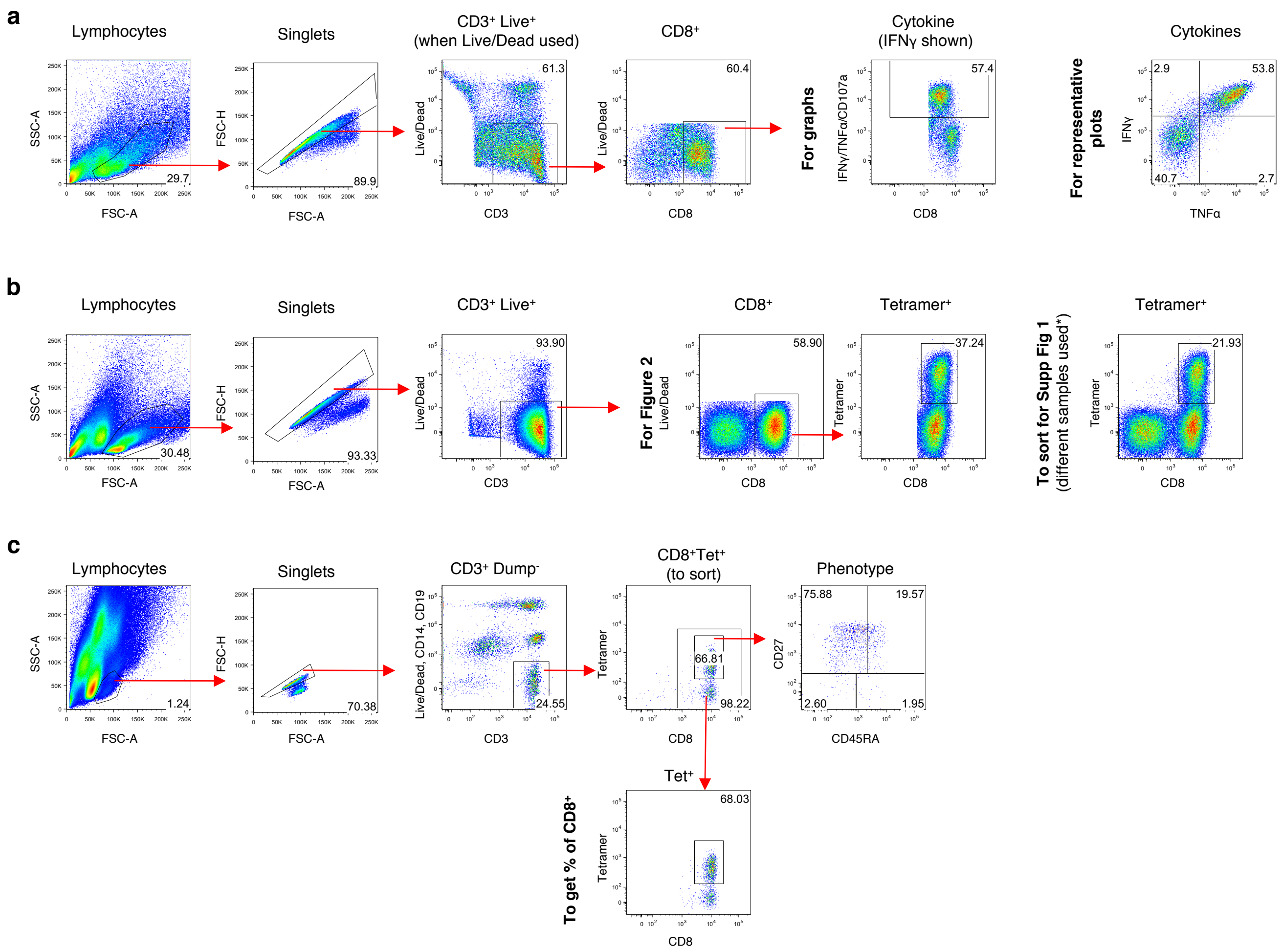
a
Lymphocytes
Singlets
CD3+ Live+ (when Live/Dead used)
CD8+
Cytokine (IFNγ shown)
Cytokines
For graphs
For representative plots
b
Lymphocytes
Singlets
CD3+ Live+
CD8+
Tetramer+
Tetramer+
For Figure 2
To sort for Supp Fig 1 (different samples used*)
c
Lymphocytes
Singlets
CD3+ Dump-
CD8+Tet+ (to sort)
Phenotype
Tet+
To get % of CD8+

Supplementary Figure 2. Gating strategies used for this study

(a) Gating strategy used for ICS of epitope-specific $CD8^+$ T cell lines as used in Figures 1 and 2. **(b)** Gating strategy used for tetramer staining of epitope-specific $CD8^+$ T cell lines as used in Figure 2 and Supplementary Figure 1. * FACS plot shown was not from a sorted sample and is indicative of the gating strategy only. **(c)** Gating strategy used for sorting of epitope-specific $CD8^+$ T cells directly ex vivo following magnetic enrichment as used in Figures 4, 5 and 8.

Supplementary Table 1. **Thermal stability of pHLA complexes**

HLA-peptide complex	Tm (°C)
HLA-B*37:01-NP_{338}	61.6 ± 0.8
HLA-B*37:01-NP_{338}-L7S	61.7 ± 1.2
HLA-B*37:01-NP_{338}-V6L	57.6 ± 1.1
HLA-B*18:01-NP_{338}	55.7 ± 1.2
HLA-B*44:05-NP_{338}	47.5 ± 0.5

Tm, or thermal melt, is the temperature required to reach 50% of unfolded protein.

Supplementary Table 2. Data Collection and Refinement Statistics

Data Collection Statistics	HLA-B*37:01-NP_{338}	HLA-B*37:01-NP_{338}-L7S	HLA-B*37:01-NP_{338} V6L	HLA-B*18:01-NP_{338}	HLA-B*44:05-NP_{338}	EM2 TCR-HLA-B*37:01-NP_{338}
Space group	$P2_12_12_1$	$P2_12_12_1$	$P2_12_12_1$	$P2_12_12_1$	$P2_12_12_1$	$P2_12_12_1$
Cell Dimensions (a,b,c) (Å)	50.88, 81.79, 110.19	50.97, 81.72, 110.56	50.86, 82.04, 110.20	50.83, 81.55, 110.69	82.19, 110.52, 50.90	46.81, 97.92, 185.59
Resolution (Å)	46.19 – 1.31 (1.33 – 1.31)	81.72 –1.50 (1.58 – 1.50)	82.04 – 1.55 (1.63 – 1.55)	46.19 – 1.21 (1.23 – 1.21)	46.23 – 1.35 (1.42 – 1.35)	47.40 – 3.00 (3.16 – 3.00)
Total No. of obs.	802119 (113954)	507353 (78130)	453643 (65126)	984959 (40588)	696389 (93213)	131903 (19319)
No. of unique obs.	111938 (16061)	73354 (10768)	67762 (9785)	140506 (6636)	101079 (13835)	18278 (2614)
Multiplicity	7.2 (6.9)	6.9 (7.3)	6.7 (6.7)	7.0 (6.1)	6.9 (6.7)	7.2 (7.4)
Data completeness (%)	99.8 (99.1)	98.3 (100.0)	100.0 (100.0)	99.6 (95.8)	98.8 (93.9)	100.0 (100.0)
I/σ_I	13.3 (3.4)	6.9 (2.3)	9.7 (2.1)	11.8 (2.4)	10.8 (2.4)	6.3 (2.0)
R_{pim}^a (%)	3.7 (30.5)	6.6 (30.2)	8.0 (42.8)	3.8 (30.5)	3.8 (30.4)	13.4 (39.1)
**Refinement Statistics**						
R_{factor}^b (%)	17.17	21.92	18.27	19.20	19.17	21.84
R_{free}^b (%)	19.27	25.06	20.67	21.36	21.05	30.12
rmsd from ideality						
Bond lengths (Å)	0.010	0.010	0.005	0.010	0.010	0.008
Bond angles (°)	1.02	1.04	0.97	1.06	1.03	1.03
Ramachandran plot (%)						
Allowed region	98.5	98.2	98.2	98.5	98.4	90.4
Disallowed region	0	0.2	0.80	0	0	1.8

[a]$R_{p.i.m} = \Sigma_{hkl} [1/(N-1)]^{1/2} \Sigma_i | I_{hkl, i} - \langle I_{hkl}\rangle | / \Sigma_{hkl} \langle I_{hkl}\rangle$. [b] $R_{factor} = \Sigma_{hkl} || F_o | - | F_c || / \Sigma_{hkl} | F_o |$ for all data except ≈ 5% which were used for R_{free} calculation. Values in parentheses are for the highest resolution-shell.

Supplementary Table 3. Donors used in this study and their HLA profiles

	Donor	HLA-A	HLA-B
**HLA-B*37:01**	Donor 1	01:01, 23:01	40, **37:01**
	Donor 2	01:25, 11:01	13:02, **37:01**
	Donor 3	01:01, 68:01	35:03, **37:01**
	Donor 4	01:01, 01:01	08:01, **37:01**
	Donor 5	01:01, 24:02	08:01, **37:01**
**HLA-B*18:01**	Donor 6	02:05, 25	**18:01 44:02**
	Donor 7	29:02, 30:02	**18:01**, **44:03**
	Donor 8	02:01, 03:02	**18:01**, 35:08
	Donor 9	02, 25:01	07:02, **18:01**
	Donor 10	02:01, 02:01	15, **18:01**
**HLA-B*44:02**	Donor 11	02:01, 11:01	35:03, **44:02**
	Donor 12	02:01, 29:02	140:2, **44:02**
	Donor 13	03:01, 32:01	**44:02, 44:02**
	Donor 14	03:01, 24:02	35:03, **44:02**
	Donor 15	02:01, 02:01	07:02, **44:02**
**HLA-B*44:03**	Donor 16	03:01, 11:01	07:02, **44:03**
	Donor 7	29:02, 30:02	**18:01**, **44:03**
	Donor 17	02:01, 32:01	07:02, **44:03**
	Donor 18	01:01, 26:01	08:01, **44:03**
	Donor 19	02, 24:02	13:02, **44:03**
**HLA-A*01:01**	Donor 1	01:01, 23:01	40, **37:01**
	Donor 20	01:01, 24:02	08:01, 57:01
	Donor 21	01:01, 31:01	07:02, 35:01

Alleles of interest are shown in bold.

Supplementary Table 4. TCRαβ repertoire usage for the recognition of NP_{338}-variant peptides

									Frequency								
									D1			D2			D3		
Code	TRAV	TRAJ	CDR3α	Length	TRBV	TRBJ	CDR3β	Length	WT	L7S	V6L	WT	L7S	V6L	WT	L7S	V6L
AA	14	43	CAMRGSGMNNDMRF	9	20-1	1-6	CSARAYRGYNSPLHF	10	16	38	4						
AB	29	43	CAARGGNDMRF	6	27	1-1	CASSLTGQGAFF	7	16	23	4						
AC	21	47	CAAPLEYGNKLVF	8	27	2-6	CASGRLVTGGGTGANVLTF	14	8	8							
AD	3	26	CAVRLTRDNYGQNFVF	11	14	2-1	CASSQVDYNEQFF	8	4		7						
AE	3	37	CAVRDSASGNTGKLIF	11	19	1-5	CASSWGTGGNQPQHF	10	4		4						
AF	6	27	CAHVNTNAGKSTF	8	5-5	1-3	CASSGLINGNTIYF	9	4		19						
AG	12-2	21	CAVKGRYNFNKFYF	9	19	2-7	CASSVGTGAYEQYF	9	4		4						
AH	19	50	CALSNAKTSYDKVIF	10	20-1	1-5	CSARPGQFYSNQPQHF	11	4		11						
AI	17	45	CATDFALSSGGGADGLTF	13	3	1-5	CASSLTGHNQPQHF	9		19	7						
AJ	8-3	24	CALTDSWGKFQF	7	11-2	2-5	CASTGTSGRAETQYF	10	8								
AK	3	34	CAVRDGGVLRTDKLIF	11	19	2-7	CASSISSGRTYEQYF	10	4								
AL	3	34	CAVRGVRSYNTDKLIF	11	19	2-3	CATQGTSGYTDTQYF	10	4								
AM	8-1	48	CAVKGVNFGNEKLTF	10	7-2*	2-2	CASNWGGRGNTGELFF	11	4								
AN	13-2	15	CAENRLGTALIF	7	27	1-1	CASSSTGREAFF	7	4								
AO	21	28	CAVLHGAGSYQLTF	9	27	2-2	CASSQTGRELFF	7	4								
AP	21	28	CAVLSGAGSYQLTF	9	27	2-7	CASSPTQREQYF	7	4								
AQ	26-1	24	CIVRVPGLGDSWGKLQF	12	7-2	1-5	CASSWGWRSNQPQHF	10	4								
AR	26-1	22	CIVRVGTSGSARQLTF	11	19	1-2	CASSISPSGFNGYTF	10	4								
AS	13-1	58	CAASHETSGSRLTF	9	15	1-6	CATSRDGLAGANSPLHF	12		4							
AT	21	30	CAVYRDDKIIF	6	11-2	2-7	CASSLWGRALSYEQYF	11		4							
AU	38-1	57	CAYRSATRSSLISEKLVF	13	27	1-6	CASRTSSYNSPLHF	9		4							
AV	21	15	CAVSPVNQAGTALIF	10	19	2-1	CASSLGRGVQFF	7			7						
AW	30	37	CGTERRPSNTGKLIF	10	19	2-5	CASSIPDGTQYF	7			7						
AX	3	34	CAVSVRADKLIF	7	29-1	2-1	CSVEGLAGPSYNEQFF	11			4						
AY	12-2	47	CAVNIRGPFMEYGNKLVF	13	19	1-2	CASSMLVANYGYTF	9			4						
AZ	17	45	CATDAVQSTGGGADGLTF	13	3	1-5	CASSRTGRNQPQHF	9			4						
BA	21	28	CAVLRGAGSYQLTF	9	27	2-2	CASSPTAGELFF	7			4						
BB	23	34	CAARGRYNTDKLIF	9	19	2-7	CASSILQGRSYEQYF	10			4						
BC	13	11	CAASGPAGYSTLTF	9	19	1-1	CASSIVQRTNTEAFF	10			4						
BD	36	28	CAVGTRLDGAGSYQLTF	12	19	1-1	CASTLGTVEAFF	7			4						
	38-1	35	CAFMRGFGNVLHC	8													
CA	35	41	CAGRNMNSGYALNF	9	19	1-1	CASSIGVRAFF	6				42	5	14			
CB	3	9	CAVSYTGGFKTIF	8	19	1-6	CASSMAQGARSPLHF	10				17		19			
CC	19	39	CALSAHRFENAGNMLTF	12	20	1-5	CSARDGQVYSNQPQHF	11				4		10			
CD	20	26	CAVLLPRDNYGQNFVF	11	12	2-1	CASSLAVHNEQFF	8				4		10			
CE	19	15	CALSAGGLDQAGTALIF	12	19	1-2	CASSMGRDGYTF	7				4		5			
CF	19	10	CALSGWGLTGGGNKLTF	12	4	2-3	CASSPGTAADTQYF	9				8					
CG	12-3	43	CAMSPSFMANDMRF	9	9	1-1	CASSEGTGGLNAFF	9				4					
CH	21	28	CAVLEGAGSYQLTF	9	27	2-1	CASSLTQNEQFF	7				4					
CI	21	32	CAVRSSEWGATNKLIF	11	11-2	2-1	CASSALTGNEQFF	8				4					
CJ	29	41	CAASANSGYALNF	8	27	1-5	CASSSLRVGAGPGQPQHF	13				4					
CK	36	39	CAVGSYPNAGNMLTF	10	4-1	1-5	CASSQEAGAYNQPQHF	11				4					
CL	21	58	CAVLGRTSGSRLTF	9	4-1	1-5	CASSQDMGGYNQPQHF	11					24				
CM	22	27	CAVEPTNAGKSTF	8	4-1	1-5	CASSQDQGAYNQPQHF	11					19				

CN	41	43	CAAPSNDMRF	5	4-1	1-6	CASSQDLGSYNSPLHF	11					14				
CO	1-2	13	CAVRVPGYQKVTF	8	4	2-1	CASSQDPGQGYNEQFF	11					5				
CP	3	22	CAVRDGFWGSARQLTF	11	14	2-3	CASSQGPTTDTQYF	9					5				
CQ	8	5	CAVRVLDTGRRALTF	10	19	1-2	CASSPYTDRQENYGYTF	12					5				
CR	12-2	22	CAPGMASGSARQLTF	10	20-1	1-6	CSAREPGRQFAFAALHF	12					5				
CS	17	50	CATPPKTSYDKVIF	9	27	1-6	CASSLLKGTLHF	7					5				
CT	21	27	CAVLPNTNAGKSTF	9	4-1	1-5	CASSQDAGLYNQPQHF	11					5				
CU	27	20	CAGPYSNDYKLSF	8	27	2-3	CASSIYRRRQGALAGPDTQYF	16					5				
CV	38-2	57	CAYRSADRLRRGSEKLVF	13	27	1-6	CASRPPHYNSPLHF	9					5				
CW	17	57	CATVPALTQGGSEKLVF	12	27	2-6	CASSMLGVLRGANVLTF	12						10			
CX	38-1	28	CAFMKHRLNSGAGSYQLTF	14	27	2-2	CASSSLAVPELFF	8						10			
CY	26-1	42	CIVRVAGYGGSQGNLIF	12	19	1-6	CASSIGSGMNSPLHF	10						5			
CZ	8-6	6	CAVRDASGGSYIPTF	10	4-1	2-7	CASSLIEGLSEQYF	9						5			
DA	21	15	CAASSVRQAGTALIF	10	19	2-1	CASSIGRSEQFF	7						5			
DB	41	45	CAVKGSYSGGGADGLTF	12	7-2	2-2	CASSLRSGTGELFF	9						5			
DC	41	45	CAVRVTGGADGLTF	9	7-2	2-2	CASSQRSGTGELFF	9						5			
	29	28	CAAISPGAGSYQLTF	10													
EA	23	52	CAATYTAGGTSYGKLTF	12	27	1-2	CASTNYAGGDRGYTF	10							11	38	12
EB	21	28	CAGLRGAGSYQLTF	9	27	2-4	CASSATKNIQYF	7							5	8	6
EC	14	4	CAMREVSGGYNKLIF	10	9	2-3	CASSVVATGGRGTDTQYF	13							5	8	
ED	8-3	34	CAVGARETYNTDKLIF	11	19	1-6	CASSPRQGNNSPLHF	10							11		6
EE	30	13	CGTERSGGYQKVTF	9	19	1-1	CASSMSAMGTEAFF	9							16		35
EF	9-2	24	CALRTDSWGKLQF	8	12-5	2-5	CASGPTGGAAETQYF	10							21		
EG	12-2	24	CAVTIGWGKLQF	7	29-1	1-1	CSVYLNTEAFF	6							5		
EH	12-3	37	CAMSALRSPSNTGKLIF	12	19	2-5	CASSMGTGPRETQYF	10							5		
EI	12-3	21	CAMKGPYNFNKFYF	9	19	1-2	CASSIGTGGNYGYTF	10							5		
EJ	13-1	11	CAATLGSGYSTLTF	9	19	1-5	CASSMGTYSNQPQHF	10							5		
EK	35	58	CAGQLPPWETSGSRLTF	12	19	1-6	CASSRLQGANSPLHF	10							5		
EL	19	33	CALSEAGSNYQLIW	9	5-6	1-1	CASSLGTPTEAFF	8							5		
	13-1	9	CAARGGFKTIF	6													
EM	36	22	CAVGTGLSGSARQLTF	11	27	2-1	CASSPTRLVSSYNEQFF	12								8	
	23	40	CAARKATPSGTYKYIF	11													
EN	34	40	CGAVLSGTYKYIF	8	5-8	2-7	CASSLGPTTYKSSYEQYF	13								8	
EO	17	45	CATVPLLTSGGGADGLTF	13	3	1-5	CASSPTGRVQPQHF	9								8	
EP	4	5	CLVGHFMDTGRRALTF	11	27	2-5	CASRSLAEKGETQYF	10								4	
EQ	21	28	CAVLEGAGSYQLTF	9	27	1-1	CASSLAQGKAFF	7								4	
ER	29	32	CAASEYGGATNKLIF	10	27	1-6	CASSPLHLRDGPKTPLHF	13								4	
ES	30	57	CGTITQGGSEKLVF	9	9	2-1	CASSVGLAEPLDEQFF	11								4	
ET	30	49	CGTENTGNQFYF	7	11-2	2-5	CASSLGPGKSSKTQYF	11								4	
EU	21	17	CAVLPIKAAGNKLTF	10	19	1-1	CASSGVRATEAFF	8									24
EV	14	49	CAMREGGNQFYF	7	11-2	2-1	CASSLVPGNHNEQFF	10									6
EW	20	30	CAVQAMGDKIIF	7	11-2	2-1	CASSLLAGYNEQFF	9									6
EX	27	21	CAGENTYNFNKFYF	9	14	1-2	CASSQLPRGGYTF	8									6
								# of sequences	**25**	**26**	**27**	**24**	**21**	**21**	**19**	**24**	**17**
								Frequency of cross-reactivity	**60**	**88**	**59**	**71**	**5**	**57**	**47**	**54**	**59**

TRAV represents the variable α-chain gene usage; TRBV represents the β-chain gene usage; frequencies in red represent universal cross-reactive TCRαβ clonotypes whilst other cross-reactive TCRαβ clonotypes are in blue. Each clonotype frequency is

expressed as the proportion of total sequences. The EM2 clonotype, from Donor 4, is shaded in blue.

Supplementary Table 5. Contact table of the EM2 TCR with HLA-B37-NP_{338}

TCR gene	TCR residue	MHC residue	Bond type
CDR1α	Tyr30	Glu154, Gln155, Ala158	VDW
CDR2α	Leu57	Arg151, Glu154	VDW
CDR2α	Lys58	Arg151	VDW
CDR3α	Gly111	Arg62	VDW
CDR3α	Tyr112-OH	Glu58-Oε1, Arg62	VDW, HB
CDR1β	Asn28	Lys146	VDW
CDR1β	Asp30	Glu76	VDW
CDR2β	Gln57	Thr69	VDW
CDR2β	Ile58	Thr69, Gln72, Thr73, Glu76	VDW
CDR2β	Val59	Glln72, Glu76, Arg79	VDW
CDR2β	Asn60-Nδ2	Gln72-Oε1, Arg75	VDW, HB
CDR2β	Asp61-Oδ2-Oδ1	Lys68-O, Thr69-Oγ1	VDW, HB
FWβ	Gln67	Gln65	VDW
FWβ	Lys83-Nζ	Glu76-Oε1-Oε2	VDW, SB
CDR3β	Met109	Lys146	VDW
CDR3β	Ser110	Gln155	VDW
CDR3β	Met112-O	Gln155-Nε2-Oε1	VDW, HB
CDR3β	Gly113	Gln155	VDW
**TCR gene**	**TCR residue**	**Peptide residue**	**Bond type**
CDR3α	Ser109	Asp3	VDW
CDR3α	Gly110-N	Phe1, Asp3-Oδ1-Oδ2	VDW, HB
CDR3α	Gln113-Nε2-Oε1	Asp3-Oδ1-Oδ2, Leu4	VDW, HB
CDR1β	Asp30-Oδ1-Oδ2	Leu7, Ser8-Oγ	VDW, HB
CDR2β	Gln57	Leu4	VDW
CDR3β	Met109	Leu7	VDW
CDR3β	Ser110	Val6, Leu7	VDW

VDW: Van der Waals interaction (cut-off at 4 Å), HB: hydrogen bond (cut-off at 3.5 Å), SB: salt bridge (cut-off at 5 Å).

Supplementary Table 6. **Surface plasmon resonance (SPR) of the EM2 TCR for NP_{338} and variants**

**pHLA complex**	**K_{deq} (µM)**	**k_{on} ($x10^4 M^{-1}s^{-1}$)**	**k_{off} (s^{-1})**	**K_{dcal} (µM)**
HLA-B*37:01-NP_{338}	133.0 ± 9.0	ND	ND	ND
HLA-B*37:01-NP_{338}-L7S	150.5 ± 14.5	ND	ND	ND
HLA-B*37:01-NP_{338}-V6L	32.3 ± 0.2	1.046 ± 0.0064	0.214 ± 0.004	20.8 ± 1.5
HLA-B*18:01-NP_{338}	NB	ND	ND	ND
HLA-B*44:05-NP_{338}	NB	ND	ND	ND

The equilibrium dissociation constant (Kd_{eq}) values represent the mean ± sem (standard error from the mean) from at least two independent experiments carried in duplicate. NB, no binding observed at the maximum concentration tested; ND, not determined.

Supplementary Table 7. Conservation of NP_{44} epitope in distinct IAV strains

		IAV - H1N1			IAV - H3N2			Other IAV Strains		
**Sequence**	**Abbreviation**	**All**	**Aust**	**Vacc**	**All**	**Aust**	**Vacc**	**pH1N1 All**	**H5N1 All**	**H7N9 All**
CTELKLSDY	WT	79.7	67.4	33.3	34.5	35.2	75.0	98.3	84.5	
CTELKL**N**DY	S7N	18.3	23.3	66.7	0.2		6.3	0.9		
CTELKLSD**H**	Y9H	0.1			63.8	64.2	18.8			
CTELKLS**E**Y	D8E	0.3						0.3		
CTELKL**T**DY	S7T	0.1			0.1			0.1		
CTEL**R**LSDY	K5R	0.2						0.2		
CT**G**LKLSDY	E3G	0.1						0.1		
CTELKL**D**DY	S7D	0.1								
CTELKL**R**DY	S7R	0.1								
CTELKLS**N**Y	D8N	0.1								
CTEL**Q**LSDY	K5Q	0.3			0.2					
CTE**I**KL**N**DY	L4I + S7N	0.1								
CTELKL**N**D**H**	S7N + Y9H	0.5	9.3		0.1					
CTEL**E**LSDY	K5E				0.1					
CTELKLSD**C**	Y9C				0.1					
CTE**I**KLSDY	L4I								1.0	
CTELKLSD**N**	Y9N				0.1				1.0	94.9
CTELKLSD**Q**	Y9Q				0.1				12.4	
CTELKL**T**D**H**	S7T + Y9H				0.2	0.6				
CTE**I**KLSD**H**	L4I + Y9H				0.2					
CTELK**F**SD**H**	L6F + Y9H				0.2					
CTELK**H**SD**H**	L6H + Y9H				0.1					
CTELKL**G**D**H**	S7G + Y9H				0.2					
CTELKL**I**D**H**	S7I + Y9H				0.1					
CTELKLS**GH**	D8G + Y9H				0.1					
CTELKL**T**D**Q**	S7T + Y9Q								1.0	
CTELKLSD**S**	Y9S									2.6
CTELKL**N**D**N**	S7N + Y9N									2.6
	Number of sequences	***1155***	***43***	***9***	***1264***	***159***	***16***	***858***	***97***	***39***
	Coverage (%)	***98.0***	***90.7***	***100.0***	***34.6***	***35.2***	***81.3***	***99.2***	***84.5***	***0.0***

Sequences were obtained from the NCBI Influenza Research Database https://www.ncbi.nlm.nih.gov/genomes/FLU/Database/nph-select.cgi?go=database. Full-length sequences of Australian (denoted Aust), vaccine (denoted Vacc) and pH1N1, H5N1 and H7N9 were obtained and were aligned using the influenza database. Underlined are anchor residues for HLA-B*37:01, and mutations are shown in bold. Shaded rows represent sequences chosen for further analysis.

Supplementary Table 8. TCRαβ repertoire usage for the recognition of NP_{44}-variant peptides

							Frequency				
							D1		D20		D21
Code	TRAV	TRAJ	CDR3α	TRBV	TRBJ	CDR3β	WT	S7N	WT	S7N	WT
GA	8-2	21	CVV-SDRNFNKF-YF	10-3	2-3	CAI-SESNGADTQ-YF	14	16			
GB	8-2	2-5	CVV-SDRNFNKF-YF	10-3	2-5	CAI-SESQGPGGETQ-YF	3	34			
GC	29	34	CAA-SGRTDKL-IF	9	2-1	CAS-SVESSGSPYQ-FF	3	3			
GD	39	57	CAV-DSWAGGSEKL-VF	25-1	1-2	CAS-SERGDTDLYGY-TF	10				
GE	3	15	CAL-DQAGTAL-IF	3-	2-3	CAS-RPLSGGAPDTQ-YF	10				
GF	41	49	CAV-RLLLRNQF-YF	9	2.7	CAS-SVGAGPTYEQ-YF	3				
GG	41	49	CAVRFLLGNQFYF	9	2.7	CASSVGAGPSYEQYF	3				
GH	4	30	CLV-GVRDDKI-IF	10-3	2-1	CAT-KPPGTDEQ-FF	3				
GI	5	12	CAE-TTDSSYKL-IF	28	2-7	CAS-TRQGDSYEQ-YF	3				
GJ	12-1	42	CVV-TGVHYGGSQGNL-IF	2	2-7	CAS-SESGSPSYYEQ-YF	3				
GK	12-1	39	CVV-TSGML-TF	7-6	2-2	CAS-SPGTDYTGEL-FF	3				
GL	12-3	17	CAM-IKAAGNKL-TF	27-1	1-1	CAS-SINRASEA-FF	3				
GM	13-1	35	CAA-GRKRFGNVL-HC	15	2-5	CAT-SREWPANRHQETQ-YF	3				
GN	13-1	4	CAA-TFSSGGYNKL-IF	6-5	2-1	CAS-RSGGANEQ-FF	3				
GO	13-2	23	CAE-ILYNQGGKL-IF	24-1	1.2	CAT-SDFNHGY-TF	3				
GP	14	37	CAM-REGTGKL-IF	11-2	1-2	CAS-SLEAAPGRYGY-TF	3				
GQ	19	23	CAL-MRNQGGKL-IF	5-8	2-1	CAS-SLEPQDTQ-YF	3				
GR	25	15	CAG-PNQAGTAL-IF	7-3	2-1	CAS-SLTGTGSYNEQ-FF	3				
GS	26-2	37	CIR-VGSNTGKL-IF	5-8	1-1	CAS-SLDSPPGVTEA-FF	3				
GT	29	54	CAA-SRGAQKL-VF	4-1	1-4	CAS-SHLQGSEKL-FF	3				
GU	29	41	CAA-SGYAL-NF	12	2-3	CAS-SFGGRAGDTDTQ-YF	3				
GV	38-2	34	CAY-QVHTDKL-IF	16	1-1	CAS-SQEDGFTEA-FF	3				
GW	8-2	21	CVV-SDRNFNKF-YF	10-3	2-3	CAI-SESLGADTQ-YF		9			
GX	8-2	21	CVV-SDRNFNKF-YF	10-3	2-3	CAG-RDHTDTQ-YF		9			
GY	12-2	38	CAG-GAGNNRKL-IW	4-1	1-5	CAS-SQDGISTGDQPQ-HF		9			
GZ	8-2	21	CVV-SDRNFNKF-YF	10-3	1-2	CAI-SEEGTGFYGY-TF		6			
HA	24	6	CAP-ASGGSYIP-TF	10-3	2-7	CAI-GGDGSYEQ-YF		6			
HB	8-2	21	CVV-SDRNFNKF-YF	10-3	2-5	CAI-SEFQGPGGETQ-YF		3			
HC	14	11	CAM-REESGYSTL-TF	4	1-1	CAS-SQDGAGTPFTEA-FF		3			
HD	1-2	33	CAV-VDSNYQL-IW	3	2-1	CAS-SQEGEAHNEQ-FF			5		
HE	1-2	33	CAV-RDSNYQL-IW	6-4	2-1	CAS-SDSPGVGYNEQ-FF			5		
HF	1-2	20	CAV-KDRDYKL-SF	6-4	1-5	CAS-SEGENQPQ-HF			5		
HG	1-2	33	CVL-MDSNYQL-IW	20-1	1-1	CSA-RRADTE-AFF			5		
HH	1-2	33	CAV-MDSNYQL-IW	20-1	2-7	CSA-REHREPYEQ-YF			5		
HI	1-2	33	CAV-LDSNYQL-IW	20-1	2-3	CSA-RGTSGETYTQ-YF			5		
HJ	1-2	33	CAV-TDSNYQL-IW	20-1	1-1	CSA-RTGDRTEA-FX			5		
HK	1-2	33	CVP-MDSNYQL-IW	24-1	2-2	CAT-SDRDRDTGEL-FF			5		
HL	1-2	33	CAV-KDSNYQL-IW	28	2-6	CAS-SFGRLSGANVL-TF			5		
HM	3	30	CAV-TPGDKI-IF	5-3	1-1	CAS-SFSVRDMGTEA-FF			5		
HN	6	34	CAL-DTDKL-IF	28	2-3	CAS-SLWASGKWSTDTQ-YF			5		

HO	12-2	3	CAV-NMARYSSASKI-IF	19	2-2	CAS-NPPGLAATGEL-FF			5		
HP	12-3	30	CAI-GDDKI-IF	28	2-3	CAS-SSTSGRARDTQ-YF			5		
HQ	12-3	36	CAM-EGGANNL-FF	19	1-2	CAS-TSVMTDSIYGY-TF			5		
HR	13-2	26	CAE-KKDNYGQNF-VF	6-8	2-1	CAS-SYLGTSAPIDEQ-FF			5		
HS	19	58	CALSSKETSGSRLTF	4-2	1-5	CAS-SRTGEHQPQ-HF			5		
HT	19	45	CAL-SDPYSGGGADGL-TF	7-9	2-5	CAS-SLAGGTGETQ-YF			5		
HU	19	11	CAL-SDGYSTL-TF	27	2-6	CAS-SLPGANVL-TF			5		
HV	19	49	CAL-SLDGNQF-YF	27	2-1	CAS-RLGESSYNEQ-FF			5		
HW	29	35	CAA-SERLGFGNVL-HC	5	2-2	CAS-SFLTGEL-FF			5		
HX	6	20	CAL-DGDYKL-SF	3	1	CAS-SQDNPYGANTEA-FF				59	
HY	8	21	CVV-SDRNFNKF-YF	10-3	2-7	CAI-SESIGLDEQ-YF				24	
HZ	8	21	CVV-SDRNFNKF-YF	10-3	2-7	CAI-SGWTGEGSEQ-YF				6	
IA	1-2	33	CAV-RNSNYQL-IW	6-4	2-3	CAS-SATGDATDTQ-YF				6	
IB	19	12	CAL-EMDSSYKL-IF	20	-5	CSA-GGLAGEETQ-YF				6	
IC	1-2	33	CAV-NNSRL-IW	20-1	2-7	CSA-RMVSYEQ-YF					5
ID	3	16	CAV-RLLDGQKL-LF	7-6	2-1	CAS-SRGTYNEQ-FF					5
IE	4	31	CLV-GDGARL-MF	28	2-7	CAS-SSSGYEQ-YF					5
IF	5	23	CAE-TPQGGKL-IF	9	2-7	CAS-SVGRGSGEQY-F					5
IG	8-6	43	CAG-GGHNNDM-RF	30	2-7	CAY-LTGFEQ-YF					5
IH	13-2	26	CAV-SVVYGQNF-VF	30	2-3	CAW-SPTSGRDTQ-YF					5
II	17	45	CAT-ALYSGGGADGL-TF	19	1-1	CAS-SILGWSEA-FX					5
IJ	19	31	CA-LSEARL-MF	4	2-1	CAS-SHLLAAYNEQ-FF					5
IK	19	39	CAR-DNAGNML-TF	6-6	2-1	CAS-SYSAWNEQ-FF					5
IL	19	45	CAL-TELGGGADGL-TF	30	2-1	CAW-SALAGGPSNEQ-FF					5
IM	20	39	CAV-QARNNAGNML-TF	19	2-7	CAS-SIVKLAGGTYEQ-YF					5
IN	21	54	CAV-WIQGAQKL-VF	11-3	2-3	CASS-LRTRTDTQ-YX					5
IO	22	32	CAV-YSRGATNKL-IF	6-1	1-5	CAS-SVGRDSSGNQPQ-HF					5
IP	25	42	CAG-SIMNYGGSQGNL-IF	9	2-7	CAS-SVSSGLYYEQ-YF					5
IQ	27	42	CAG-DLSYGGSQGNL-IF	2	2-7	CAS-SWTSGRPEQ-YF					5
IR	29	45	CAA-TGGGADGL-TF	9	2-7	CAS-SGASYEQ-YF					5
IS	35	45	CAG-YSGGGADGL-TF	5-8	2-5	CAS-SPMTGDGTQ-YF					5
IT	38-1	54	CAF-LPGGAQKL-VF	25-1	2-3	CAS-SVRADTQ-YF					5
IU	41	53	CAV-SSFNSGGSNYKL-TF	6-4	2-1	CAS-SDSSFGVGLGNEQ-FF					5
						# of sequences	**29**	**32**	**20**	**17**	**19**

TRAV represents the variable α-chain gene usage; TRBV represents the β-chain gene usage; sequences in blue residues represent conserved CDR3α chains. Each clonotype frequency is expresses as the proportion of total sequences.

Supplementary Table 9. Primers used in this study

	Primer sequence 5'-3'	
**TRAV**	**External**	**Internal**
**1**	AACTGCACGTACCAGACATC	GCACCCACATTTCTKTCTTAC
**2**	GATGTGCACCAAGACTCC	CACTCTGTGTCCAATGCTTAC
**3**	AAGATCAGGTCAACGTTGC	ATGCACCTATTCAGTCTCTGG
**4**	CTCCATGGACTCATATGAAGG	ATTATATCACGTGGTACCAACAG
**5**	CTTTTCCTGAGTGTCCGAG	TACACAGACAGCTCCTCCAC
**6**	CACCCTGACCTGCAACTATAC	TGGTACCGACAAGATCCAG
**7**	AGCTGCACGTACTCTGTCAG	ACAATTTGCAGTGGTACAGG
**8-1**	CTCACTGGAGTTGGGATG	GTCAACACCTTCAGCTTCTC
**8-3**	CACTGTCTCTGAAGGAGCC	TTTGAGGCTGAATTTAAGAGG
**8-2 & 4**	GCCACCCTGGTTAAAGG	AGAGTGAAACCTCCTTCCAC
**8-6**	GAGCTGAGGTGCAACTACTC	AACCAAGGACTCCAGCTTC
**8-7**	CTCTGGAACTGAAGTGCAAC	GGAGTTCCTTCTCTCTTCTGG
**9-1 & 2**	TGGTATGTCCAATATCCTGG	GAAACCACTTCTTTCCACTTG
**10**	CAAGTGGAGCAGAGTCCTC	GAAAGAACTGCACTCTTCAATG
**12-1 & 3**	CARTGTTCCAGAGGGAGC	AAGATGGAAGGTTTACAGCAC
**13-1**	CATCCTTCAACCCTGAGTG	TCAGACAGTGCCTCAAACTAC
**13-2**	CAGCGCCTCAGACTACTTC	CAGTGAAACATCTCTCTCTGC
**14**	AAGATAACTCAAACCCAACCAG	AGGCTGTGACTCTGGACTG
**16**	AGTGGAGCTGAAGTGCAAC	GTCCAGTACTCCAGACAACG
**17**	GGAGAAGAGGATCCTCAGG	CCACCATGAACTGCAGTTAC
**18**	AGAAAACCAGGAGACGGAC	CAGGCCAGTCCTATCAAGAG
**19**	AGGTAACTCAAGCGCAGAC	TGTGACCTTGGACTGTGTG
**20**	CACAGTCAGCGGTTTAAGAG	TCTGGTATAGGCAAGATCCTG
**21**	TTCCTGCAGCTCTGAGTG	AACTTGGTTCTCAACTGCAG
**22**	GTCCTCCAGACCTGATTCTC	CTGACTCTGTGAACAATTTGC
**23**	TGCTTATGAGAACACTGCG	TGCATTATTGATAGCCATACG
**24**	CTCAGTCACTGCATGTTCAG	TGCCTTACACTGGTACAGATG
**25**	GGACTTCACCACGTACTGC	TATAAGCAAAGGCCTGGTG
**26-1**	GCAAACCTGCCTTGTAATC	CGACAGATTCACTCCCAG
**26-2**	AGCCAAATTCAATGGAGAG	TTCACTTGCCTTGTAACCAC
**27**	TCAGTTTCTAAGCATCCAAGAG	CTCACTGTGTACTGCAACTCC
**29**	GCAAGTTAAGCAAAATTCACC	CTGCTGAAGGTCCTACATTC
**30**	CAACAACCAGTGCAGAGTC	AGAAGCATGGTGAAGCAC
**34**	AGAACTGGAGCAGAGTCCTC	ATCTCACCATAAACTGCACG
**35**	GGTCAACAGCTGAATCAGAG	ACCTGGCTATGGTACAAGC
**36**	GAAGACAAGGTGGTACAAAGC	ATCTCTGGTTGTCCACGAG
**38**	GCACATATGACACCAGTGAG	CAGCAGGCAGATGATTCTC
**39**	CTGTTCCTGAGCATGCAG	TCAACCACTTCAGACAGACTG
**40**	GCATCTGTGACTATGAACTGC	GGAGGCGGAAATATTAAAGAC
**41**	AATGAAGTGGAGCAGAGTCC	TTGTTTATGCTGAGCTCAGG
**Constant**	GACCAGCTTGACATCACAG	TGTTGCTCTTGAAGTCCATAG
**TRBV**	**External**	**Internal**

**2**	TCGATGATCAATTCTCAGTTG	TTCACTCTGAAGATCCGGTC
**3**	CAAAATACCTGGTCACACAG	AATCTTCACATCAATTCCCTG
**4**	TCGCTTCTCACCTGAATG	CCTGCAGCCAGAAGACTC
**5-1 & 4**	GATTCTCAGGKCKCCAGTTC	CTTGGAGCTGGRSGACTC
**5-5 & 8**	GTACCAACAGGYCCTGGGT	TCTGAGCTGAATGTGAACG
**6-1 & 3 & 5 & 9**	ACTCAGACCCCAAAATTCC	GTGTRCCCAGGATATGAACC
**6-4**	ACTGGCAAAGGAGAAGTCC	TGGTTATAGTGTCTCCAGAGC
**7-1 & 3**	TRTGATCCAATTTCAGGTCA	TCYACTCTGAMGWTCCAGCG
**7-4 & 9**	CGSWTCTYTGCAGARAGGC	TGRMGATYCAGCGCACA
**9**	GATCACAGCAACTGGACAG	GTACCAACAGAGCCTGGAC
**10**	TGTWCTGGTATCGACAAGACC	TCCYCCTCACTCTGGAGTC
**11**	CGATTTTCTGCAGAGACGC	GACTCCACTCTCAAGATCCA
**12**	ARGTGACAGARATGGGACAA	CYACTCTGARGATCCAGCC
**13**	AGCGATAAAGGAAGCATCC	CATTCTGAACTGAACATGAGC
**14**	CCAACAATCGATTCTTAGCTG	ATTCTACTCTGAAGGTGCAGC
**15**	AGTGACCCTGAGTTGTTCTC	ATAACTTCCAATCCAGGAGG
**16**	GTCTTTGATGAAACAGGTATGC	CTGTAGCCTTGAGATCCAGG
**17**	AGTTGCTGATTTCCTTCCAG	CACGCTGAAGATCCATCC
**18**	CATAGATGAGTCAGGAATGCC	CGATTTTCTGCTGAATTTCC
**19**	AGTTGTGAACAGAATTTGAACC	TTCCTCTCACTGTGACATCG
**20**	AAGTTTCTCATCAACCATGC	ACTCTGACAGTGACCAGTGC
**23**	GCGATTCTCATCTCAATGC	GCAATCCTGTCCTCAGAAC
**24**	CCTACGGTTGATCTATTACTCC	GATGGATACAGTGTCTCTCGA
**25**	ACTACACCTCATCCACTATTCC	CAGAGAAGGGAGATCTTTCC
**27**	TGGTATCGACAAGACCCAG	TTCYCCCTGATYCTGGAGTC
**29**	TTCTGGTACCGTCAGCAAC	TCTGACTGTGAGCAACATGAG
**30**	TCCAGCTGCTCTTCTACTCC	AGAATCTCTCAGCCTCCAGAC
**Constant**	TAGAACTGGACTTGACAGCG	TTCTGATGGCTCAAACACAG
**Vector**	**Sense**	**Anti-sense**
	GTAAAACGACGGCCAG	CAGGAAACAGCTATGAC

TRAV represents the variable α-chain gene usage; TRBV represents the β-chain gene usage.
