# Supplementary material for: Broad CD8+ T cell cross-recognition of distinct influenza A strains in humans
Source: Nat Commun. 2018 Dec 21;9:5427. doi: 10.1038/s41467-018-07815-5 (PMC6303473; doi:10.1038/s41467-018-07815-5)
Supplement: Supplementary file 3 — Reporting Summary [file 41467_2018_7815_MOESM3_ESM.pdf]

nature research

Corresponding author(s): DBPR NCOMMS-18-22543A

Reporting Summary

Nature Research wishes to improve the reproducibility of the work that we publish. This form provides structure for consistency and transparency in reporting. For further information on Nature Research policies, see Authors & Referees and the Editorial Policy Checklist.

Statistical parameters

When statistical analyses are reported, confirm that the following items are present in the relevant location (e.g. figure legend, table legend, main text, or Methods section).

n/a	Confirmed	
☐	☒	The exact sample size (n) for each experimental group/condition, given as a discrete number and unit of measurement
☐	☒	An indication of whether measurements were taken from distinct samples or whether the same sample was measured repeatedly
☐	☒	The statistical test(s) used AND whether they are one- or two-sided<br>*Only common tests should be described solely by name; describe more complex techniques in the Methods section.*
☐	☒	A description of all covariates tested
☐	☒	A description of any assumptions or corrections, such as tests of normality and adjustment for multiple comparisons
☐	☒	A full description of the statistics including central tendency (e.g. means) or other basic estimates (e.g. regression coefficient) AND variation (e.g. standard deviation) or associated estimates of uncertainty (e.g. confidence intervals)
☐	☒	For null hypothesis testing, the test statistic (e.g. F, t, r) with confidence intervals, effect sizes, degrees of freedom and P value noted<br>*Give P values as exact values whenever suitable.*
☒	☐	For Bayesian analysis, information on the choice of priors and Markov chain Monte Carlo settings
☒	☐	For hierarchical and complex designs, identification of the appropriate level for tests and full reporting of outcomes
☒	☐	Estimates of effect sizes (e.g. Cohen's d, Pearson's r), indicating how they were calculated
☐	☒	Clearly defined error bars<br>*State explicitly what error bars represent (e.g. SD, SE, CI)*

Our web collection on statistics for biologists may be useful.

Software and code

Policy information about availability of computer code

Data collection	All software used for collection and analysis listed below
Data analysis	Prism 6 (GraphPad, CA, USA)<br>BIAevaluation Version 3.1<br>Pymol 1.7.3.3<br>CCP4 6.5.000<br>XDS 2015<br>Buster 2016<br>Flowjo V9.7.6 - 9.9.6<br>Finch TV V1.5.0

For manuscripts utilizing custom algorithms or software that are central to the research but not yet described in published literature, software must be made available to editors/reviewers upon request. We strongly encourage code deposition in a community repository (e.g. GitHub). See the Nature Research guidelines for submitting code & software for further information.

Data

Policy information about availability of data

All manuscripts must include a data availability statement. This statement should provide the following information, where applicable:

- Accession codes, unique identifiers, or web links for publicly available datasets
- A list of figures that have associated raw data
- A description of any restrictions on data availability

Coordinates submitted to PDB database, and the PDB codes are 6MT6 (HLA-B*37:01-NP338), 6MT4 (HLA-B*37:01-NP338-L7S), 6MT5 (HLA-B*37:01-NP338-V6L), 6MTL (HLA-B*44:05-NP338), 6MT3 (HLA-B*18:01-NP338), and 6MTM (EM2 TCR-HLA-B*37:01-NP338). All other data that support the findings of this study are available from the corresponding author upon reasonable request. A reporting summary for this Article is available as a Supplementary Information file.

Field-specific reporting

Please select the best fit for your research. If you are not sure, read the appropriate sections before making your selection.

☒ Life sciences ☐ Behavioural & social sciences ☐ Ecological, evolutionary & environmental sciences

For a reference copy of the document with all sections, see nature.com/authors/policies/ReportingSummary-flat.pdf

Life sciences study design

All studies must disclose on these points even when the disclosure is negative.

Sample size	With limited access to human samples and immune responses being controlled by host genetics, a sample size of >3 is considered worth reporting in human immunology.
Data exclusions	No data was excluded from this manuscript
Replication	In human research using primary samples, findings/trends are considered reproducible if the conclusions draw are observed in multiple individuals with the same HLA type in this instance. Furthermore, in the instance of positive responses, these were confirmed using multiple assays including tetramer staining in vitro and ex vivo, and intracellular cytokine staining (ICS).
Randomization	In this study we were assessing CD8+ T cell responses towards the NP338 peptide by individuals with specific HLAs. As such, samples were selected and grouped based on their HLA type.
Blinding	In this study we assessed immune responses in individuals with specific HLA types, and as such, samples were specifically allocated and blinding by the investigators was not required.

Reporting for specific materials, systems and methods

Materials & experimental systems

n/a	Involved in the study	
☐	☒	Unique biological materials
☐	☒	Antibodies
☐	☒	Eukaryotic cell lines
☒	☐	Palaeontology
☒	☐	Animals and other organisms
☐	☒	Human research participants

Methods

n/a	Involved in the study	
☒	☐	ChIP-seq
☐	☒	Flow cytometry
☒	☐	MRI-based neuroimaging

Unique biological materials

Policy information about availability of materials

Obtaining unique materials	Human samples were collected under specific ethics for specific projects and as such are non transferable. Furthermore, antigen presenting cell lines were supplied by collaborators with MTAs in place as per "Eukaryotic cell lines" below.

Antibodies

Antibodies used

Antibody Colour Dilution Clone Company Catalogue #
CD107a AF488 1:100-1:200 eBioH4A3 eBioscience 53-1079-41
CD14 APC H7 1:50-1:100 MΦP9 BD 641394
CD19 APC Cy7 1:100 HIB19 Biolegend 302218
CD27 APC 1:50-1:100 L128 BD 337169
CD3 PeCy7 1:50 - 1:100 UCHT1 eBioscience 25-0038-42
CD3 PE 1:25-1:50 UCHT1 BD Pharmingen 555333
CD3 Pac Blue 1:50-1:100 UCHT1 Biolegend 300431
CD4 APC H7 1:50-1:100 SK3 BD 614398
CD45 RA FITC 1:50-1:100 HI100 BD Pharmingen 555488
CD8 APC 1:50-1:100 SK1 BD 340584
CD8 PerCP Cy5.5 1:50 SK1 BD 341051
IFNg PE 1:40 25723,11 BD 340452
IFNg PerCP Cy5.5 1:100 4S.B3 eBioscience 45-7319-41
IFNg V500 1:50-1:100 B27 BD Horizon 561980
TNFa APC 1:50-1:100 6401,1111 BD 340534
TNFa PeCy7 1:100 MAb11 BD Pharmingen 557647

Validation

All antibodies are commercially available and many have been previously published. Relevant information is available directly from the companies websites.

Eukaryotic cell lines

Policy information about cell lines

Cell line source(s)

Human derived antigen presenting cell lines (APC) were kindly provided by Prof. James McCluskey (University of Melbourne, VIC, Australia; C1R-B*44:03) and Dr. Nicole Mifsud (Monash University, VIC, Australia; C1R-B*18:01). The A2-20091204 (HLA-B*37:01+) BLCLs cells were provided by Prof. Weisan Chen (LaTrobe University, VIC, Australia).

Authentication

All cell have been previously used in the laboratory and demonstrated their ability to activate cells of the relevant HLAs. In this instance specific HLA cell lines were not available, or if their ability to activate cells was unknown, controls were completed in which peptide was placed straight into the well rather than the use of APCs (HLA-B*44:02+ donors in this study)

Mycoplasma contamination

All cell lines tested mycoplasma negative before being obtained from collaborators.

Commonly misidentified lines
(See ICLAC register)

N/A

Human research participants

Policy information about studies involving human research participants

Population characteristics

Donor information including HLA typing result is reported in Supplementary Table 3.

Recruitment

All work was undertaken in line with the Australian National Health and Medical Research Council (NHMRC) Code of Practice, with ethics approval by the University of Melbourne Human Ethics Committee, ethics numbers 0931311 and 1443389. Australian Red Cross Blood donors provided written informed consent on the day of their blood donation. Written informed consent was obtained from all healthy blood donors.

Flow Cytometry

Plots

Confirm that:

☒ The axis labels state the marker and fluorochrome used (e.g. CD4-FITC).

☒ The axis scales are clearly visible. Include numbers along axes only for bottom left plot of group (a 'group' is an analysis of identical markers).

☒ All plots are contour plots with outliers or pseudocolor plots.

☒ A numerical value for number of cells or percentage (with statistics) is provided.

Methodology

Sample preparation

Buffy coats were obtained from the Australian Red Cross Blood Service (ARCBS) and whole blood donations from healthy volunteers. PBMCs were separated using density gradient separation and were cryopreserved until use. All samples were HLA-typed by the Victorian Transplant and Immunogenetics Service (VTIS, West Melbourne, Victoria, Australia) at the ARCBS tissue-typing Laboratory. All detailed staining and assay protocols are outlines in the methods section. Different antibody panels were used throughout this manuscript, as outlines in the methods section, and as such, specific flourochromes have been omitted

from markers in figures so as not to be misleading.

Instrument: A range of instruments were used in this study including BD Canto II, BD Fortessa and BD Aria III

Software: Diva software was used for data collection. Data was analysed using Flowjo V9.7.6 - 9.9.6

Cell population abundance: Samples were single-cell sorted into PCR plates and as such, no bulk sorting was complete. However, in data collected for other projects the purity of samples was always excellent.

Gating strategy: All gating strategies are outlines in their relevant figure legends and Supplementary Figure 2.

☒ Tick this box to confirm that a figure exemplifying the gating strategy is provided in the Supplementary Information.
